# Artificial I ntelligence-Assisted Fetal Echocardiography: Improving Early Detection and Neonatal Outcomes of Congenital Heart Disease

**DOI:** 10.26502/fccm.92920488

**Published:** 2026-05-06

**Authors:** Karina Patel, Devendra K Agrawal

**Affiliations:** Department of Translational Research, College of Osteopathic Medicine of the Pacific, Western University of Health Sciences, Pomona, California 91766 USA

**Keywords:** Artificial Intelligence (AI), Congenital heart disease (CHD), Fetal echocardiography, Postnatal outcomes

## Abstract

Congenital Heart Disease (CHD) encompasses a range of structural abnormalities of the heart and great vessels present at birth and is associated with significant lifelong morbidity, including heart failure and arrhythmias. Early diagnosis is critical for optimizing perinatal management and improving outcomes. This review focuses on early fetal echocardiography, a specialized ultrasound technique that enables detection of cardiac abnormalities in utero, as early as 11–14 weeks of gestation, compared to traditional imaging performed at approximately 20 weeks. Advancements in early imaging have improved the identification of high-risk fetuses, particularly those with genetic predispositions or family history of CHD, allowing for earlier clinical decision-making and intervention planning. In select cases, prenatal detection facilitates in utero management of conditions such as aortic stenosis and hypoplastic left heart syndrome. Additionally, emerging applications of artificial intelligence (AI) have enhanced image analysis and diagnostic accuracy, supporting clinicians in the early recognition of CHD. Despite these advances, challenges remain, including variability in diagnostic accuracy in early gestation, risk of false positives, and limited access to specialized imaging technologies. Continued integration of AI-driven tools and the development of standardized screening protocols hold promise for improving diagnostic consistency and long-term outcomes in patients with CHD.

## Introduction

Congenital Heart Disease is the most common type of birth defect, affecting nearly 8 in every 1,000 births [1,2]. More recent data, through meta-analyses, show that this number has risen to around 9.4 per 1,000 births. CHD continues to contribute to neonatal morbidity and mortality, particularly in critical forms that require intervention early in life [3,4].

Prenatal detection is important because it allows delivery planning, early specialist involvement, immediate stabilization of the neonate, and transfer to cardiac centers if needed. Evidence shows that prenatal diagnosis of CHD reduces the risk of death in select infants and improves perinatal management by enabling early intervention. However, the survival benefit varies based on the type of CHD, with the most benefit being shown in critical CHD and transposition of the great arteries, while it is less consistent in other CHD types.

Fetal echocardiography has shifted prenatal cardiac assessment from a mid-trimester test to an increasingly earlier diagnostic tool [5,6]. Fetal echocardiography uses ultrasound technology to view cardiac structure and function. Using a probe that ranges from low to high frequencies, acoustic waves are transmitted that travel through the mother’s tissues and reflect and scatter. Echoes are returned that display an image with varying brightness [7]. Fetal echocardiography was first developed using M-mode in the 1960s, a one-dimensional view of structures. Following this, two-dimensional imaging was developed in the 1970s and 1980s, followed by color Doppler techniques and later three and four-dimensional techniques. Fetal echocardiography is most performed at 18-22 weeks of gestation, but improvements in ultrasound technology now allow for reliable first trimester and early second-trimester assessment in conditions such as high-risk pregnancies. Early fetal echocardiography allows for earlier counseling and care planning, although some structures may be hard to evaluate in early gestation or evolve over time. However, there are mathematical limitations of fetal echocardiography as a screening tool in the setting of a normal second-trimester ultrasound [8].

More recently, AI-based tools have emerged in all almost every area of medical practice, including its role as an adjunct tool in decision making across the preoperative, peri-operative, and postoperative stages of various surgery [9-11]. Indeed, AI has been useful in: (i) identifying biomarkers derived from neuroimaging, electrophysiology, and digital phenotyping [12], (ii) pattern recognition of electromagnetic field signals in the brain [13,14], (iii) optimization of bio-printed constructs [15], (iv) enhancing precision, reducing recovery times, and improving outcomes [7], and (v) developing prediction algorithms in personalized care and rehabilitation [11,16,17]. In the detection of CHD cases, application of AI offers a promising extension of fetal cardiac imaging to help make ultrasound systems more reliable and not miss important abnormalities in imaging. AI models have been developed that can automatically identify standard cardiac views, quantify cardiac function by measuring 3D cardiac chamber columns and ejection fraction, and support distinguishing normal from abnormal hearts. AI has the potential to improve detection and reduce human error, standardize image interpretation, reduce operator dependence, and expand access to effective fetal cardiac screening. However, limitations do exist, including dependence on high-quality labeled data, variability in image acquisition, and ethical concerns that must be addressed before AI is widely clinically implemented [1,18-26].

## Developmental Differences in Fetal Heart Across Gestation

During embryogenesis, the heart is one of the first organs formed. Throughout fetal development, the heart undergoes a transition from rapid structural formation in the first trimester to growth and maturation in the second trimester. In early gestation, cardiac development is driven by progenitor cells derived from the mesoderm, proepicardium, and neural crest. These cells are specially called first heart field (FHF) and second heart field (SHF) progenitor cells, cardiac neural crest cells, and proepicardial progenitor cells. Using signaling cells and gene networks, these cells are specified and differentiated, and together, establish the primitive heart tube and give rise to the four-chambered structure through looping, septation, and outflow tract formation. If problems in these signaling cells and gene networks arise, this can cause congenital heart defects in the fetus [27].

By the late first trimester, around 10-13 weeks, most cardiac structures, including the atria, ventricles, valves, and great vessel relationships, are already formed and spatially organized. However, at this stage, the myocardium is relatively immature, ventricular compliance is limited, and inflow patterns are still evolving, which reflects incomplete maturation despite almost complete anatomic formation.

Transitioning to the early second-trimester, around 14-20 weeks, more refinement and maturation occur. This includes thickening of the myocardium, improvement of ventricular compliance, maturation of diastolic filling patterns, and continuous remodeling of the conduction system and valves. Imaging quality during this phase also improves due to larger fetal size and more acoustic windows, which allows for more detailed assessment of the fetal heart compared to earlier gestation.

Due to this, fetal echocardiography in the first trimester is limited by incomplete visualization of cardiac structures, as demonstrated by variable detection rates across gestational stages [28]. Basic structures such as ventricular inflow can be identified, but complex features such as atrioventricular valves and pulmonary veins remain difficult to assess prior to 12-13 weeks. This reflects both the small size and ongoing developmental maturation of the fetal heart during this stage. However, in the early second-trimester, visualization improved substantially due to the increasing fetal size and structural maturation, allowing more consistent identification of views, including the four-chamber and outflow tract views. However, limitations may still exist for smaller or more complex structures.

## Early and very early fetal echocardiography

Due to improved ultrasound resolution and better understanding of how the fetal heart develops, early fetal echocardiography is now a valuable tool in diagnosing CHD. Traditionally, the optimal timing for a comprehensive fetal echocardiogram is 18-22 weeks of gestation, in the mid-second trimester, when cardiac structures can be visualized. Early fetal echocardiography is around 11-15 weeks, in the late first and early second-trimester, which allows for earlier detection in high risk-pregnancies. Very early fetal echocardiography is performed in the late first trimester, around 10-13 weeks of gestation. Prenatal diagnosis through fetal echocardiography plays a critical role in perinatal planning and clinical-decision making. This early identification of CHD allows for delivery at care centers with pediatric cardiac expertise, immediate postnatal stabilization and intervention, additional prenatal testing, and informed decision-making for expecting parents regarding managing their pregnancy.

Very early and early fetal echocardiography are indicated in high-risk scenarios and are not routine for all pregnancies. These high-risk scenarios include prior children with CHD, known maternal CHD, maternal autoimmune disease, increased nuchal translucency (indicating chromosomal abnormalities) or abnormal early screening, and suspicion of cardiac abnormality on early ultrasound.

Even when fetal echocardiography is performed, repeat evaluation in the second trimester at 18-22 weeks of gestation is necessary. Early scans have lower sensitivity due to the small heart size and ever-changing anatomy of the fetus. Additionally, not all cardiac abnormalities are apparent in early gestation and can develop at later stages. Overall, visualization of key structures is more reliable at 18-22 weeks. Therefore, it is recommended that all patients who undergo early fetal echocardiography should also still receive a standard mid-second-trimester study to confirm anatomy and avoid missed diagnoses [29].

## Imaging Challenges in Early Gestation

Imaging in early gestation is limited by both technical and developmental factors. The small size of the fetal heart and rapid heart rate, ranging from 110-160 beats per minute, in the first trimester reduces spatial and temporal resolution, making obtaining detailed cardiac views more challenging [27]. Some features, such as ventricular inflow, can be identified as early as 10 weeks, while more complex structures, such as the four-chamber view and pulmonary veins, are not reliably visualized until 12-13 weeks. Some structures are detected in less than half of cases, even utilizing Doppler imaging. These limitations lead to diagnostic uncertainty in early fetal echocardiography. Despite these challenges, early imaging provides important clinical value by allowing for early detection of structural cardiac anomalies and enabling improved perinatal planning and management [28].

## Maternal and Fetal Factors Affecting Image Quality

Maternal factors such as body mass index (BMI), uterine fibroids, and abdominal scars can affect fetal cardiac imaging [29]. As BMI increases, there is a particularly reduced visualization through fetal echocardiography and poor image quality. Patients with obesity, or a BMI greater than or equal to 30 kg/m^2, demonstrate lower image resolution [30]. This reduction is primarily attributable to increased soft tissue thickness, which weakens ultrasound transmission and the clarity of returning echoes. Additionally, large fibroids are likely to develop during pregnancy, which are essentially tumors of the uterus. Fibroids have been shown to reduce image quality in transabdominal imaging by distorting uterine anatomy and blocking ultrasound pathways, limiting optimal visualization [31]. Finally, abdominal scars have been associated with interfering with the ultrasound transmission pathway. To overcome this interference, studies have shown that special adjustments such as changing angles and avoiding the scar in ultrasound are necessary to achieve a proper view [32]. Fetal factors such as gestational age, position, and distance from the ultrasound probe also contribute to variability in image acquisition. Poor positioning of the fetus can reduce diagnostic accuracy and block views, such as when the spine is in front [33]. Overall, a variety of maternal and fetal factors contribute to a change in image quality.

## Imaging Approaches in Early Fetal Echocardiography

Fetal echocardiography encompasses multiple imaging approaches, primarily including transabdominal and transvaginal ultrasound techniques that are used to evaluate fetal cardiac anatomy at different gestation stages. In early fetal echocardiography, both techniques may be used, each with its own advantages and limitations. Transvaginal imaging provides spatial resolution in early gestation, between 11 and 13 weeks, allowing improved visualization of small and developing heart structures. However, it is limited due to a restriction in the field of view. In contrast, transabdominal echocardiography has a broader field of visualization and is more effective later in development, as gestational age increases. It allows for the detection of CHDs as early as 12-15 weeks, although small abnormalities are still hard to identify. To optimize diagnostic accuracy in early gestational stages, both techniques are used together [34].

## Detection of Major Congenital Heart Defects in Early Gestation

Early fetal echocardiography performed in the late first to early second-trimester enables detection of many congenital heart diseases (CHDs), particularly severe structural abnormalities that involve cardiac chambers and outflow tracts. At this stage, there is high sensitivity of fetal echocardiography for major CHDs. Another study showed that first-trimester fetal echocardiography can identify specific major CHDs through Doppler flow patterns, but further validation is necessary to achieve fully accurate detection [35]. These major CHDs include hypoplastic left heart syndrome, tetralogy of Fallot, transposition of the great arteries, truncus arteriosus, double outlet right ventricle, and atrioventricular septal defects, all of which produce significant structural or outflow tract abnormalities that can be identifiable in early fetal echocardiography. However, this ability to detect these abnormalities is not uniform across all lesion types [36]. Mild defects, such as small ventricular septal defects, are often missed due to limited resolution, while defects such as coarctation of the aorta, pulmonary venous abnormalities, and partial atrioventricular septal defects may be too hard to visualize in the super early stages of gestation. Therefore, early fetal echocardiography should be used in conjunction with standard second-trimester evaluation, not as a replacement, in cases of all CHDs. Follow-ups are always necessary to identify lesions that were not apparent or fully developed at the time of early assessment.

### Artificial Intelligence (AI) in Fetal Echocardiography

Artificial Intelligence (AI) is emerging as a powerful tool in fetal echocardiography, with applications in diagnosing congenital heart disease and improving image quality [26]. AI-based models, particularly those using deep learning, have demonstrated high sensitivity (>90%) in detecting fetal CHDs. AI has been shown to outperform traditional screening approaches and offers potential as an effective early screening tool. AI systems can automatically identify structural abnormalities, classify cardiac defects, and assist physicians in interpreting echocardiographic data. Additionally, AI contributes to image enhancement and quality, improving visualization of cardiac structures through techniques such as automated image processing, segmentation, and noise reduction. This automation is valuable in fetal imaging, as small anatomical size, motion, and operator dependence can negatively impact the quality of the image produced. AI can also help to reduce human error and variability between different ultrasound technicians, “standardizing” image acquisition. Although AI has all these advantages, limitations such as data availability, clinical workflow integration, and ethical considerations remain. More growth and maturation in AI is needed for its widespread implementation [7].

## Safety Considerations of Early and Very Early Echocardiography

Early and very early fetal echocardiography provides significant clinical benefits, including earlier detection of congenital heart disease and improved prenatal management. However, it is also important to consider the negative effects related to ultrasound exposure. Ultrasound is generally considered safe, but it interacts with biological tissues through thermal and mechanical mechanisms. This fact is relevant in early gestation, when biological, or embryonic, tissues are more sensitive [37]. As acoustic energy is absorbed from the ultrasound transducer, it is converted to heat, which can cause thermal effects. Studies have shown that the Doppler ultrasound can increase tissue temperature by approximately 2 degrees Celsius within minutes of exposure [38]. There can also be mechanical effects such as pressure changes and cavitation. However, these effects are influenced by exposure time and acoustic intensity, with longer scan durations increasing the amount of energy delivered to tissues. In fetal echocardiography, Doppler modalities are commonly used, which generate higher acoustic outputs than standard imaging. Therefore, in the first trimester, it is important to use this modality cautiously. Although no definitive harmful effects have been observed in humans, evidence supports minimizing exposure duration and adhering to safety principles when performing early fetal echocardiography.

## Impact of Early Diagnosis on Postnatal Outcomes

Prenatal diagnosis of congenital heart disease has positive, along with negative, outcomes. Early diagnosis allows for improved perinatal planning and counseling, which helps support clinical decision-making and preparation necessary for any neonatal interventions. Although there is a lot of stress associated with early diagnosis, interventions earlier on, such as therapy, can help parents manage and accept the condition of their child and lead to lower stress as time progresses.

However, an early diagnosis of CHD can be an unexpected shock to parents and have a strong psychological impact. Parents often experience increased anxiety, depression, and emotional distress following diagnosis, having a completely different view of pregnancy than what they first imagined. This stress can last throughout the pregnancy and into the postnatal period and has been linked to negative effects on fetal growth and neurodevelopmental outcomes. A mother’s stress not only has negative impacts in the short term but can also impact the child in the long term. Studies have shown that maternal stress can cause sleep disorders in children, altered emotional reactivity, ADHD symptoms, and altered cognitive function [39].

Additionally, prenatal diagnosis plays a critical role in shaping family decisions regarding continuing pregnancy and future care. Following an early diagnosis, parents have the decision to terminate the pregnancy or not, which also leads to feelings of guilt and future distress. Therefore, it is critical that parents are fully informed about their options by physicians to make an educated decision on their child’s outcome.

Overall, although prenatal diagnosis has many benefits in improving health outcomes for the child, it is important that the parents and families of patients are well cared for and supported through resources such as counseling, continued support through pregnancy from professionals and family/friends, and guidance through future decisions such as delivery, economic concerns, and the day to day life of the child [40].

## Future Directions

Future integration of early fetal echocardiography and artificial intelligence-based tools has the potential to enhance prenatal screening by automating image acquisition, standardizing cardiac views, and assisting with real-time diagnostic interpretation [25]. AI systems can help in reducing operator dependence by ensuring consistency in key views of the heart, such as four-chamber and outflow tract views. This will overall improve diagnostic accuracy and reduce variability.

Standardization of fetal echocardiography protocols is a large benefit, as uniform imaging practices can improve reproducibility across different ultrasound technicians [41]. However, there are still limitations, as successful standardization requires the creation of a large, diverse dataset. Additionally, AI has potential bias, and operators must be mindful of outside factors that may affect image quality, such as fetal position and material factors. Therefore, AI should be used as a supportive tool, rather than a replacement for a clinician’s expertise.

Future directions in fetal echocardiography also extend beyond AI and focus on improving early detection, diagnostic accuracy, and clinical outcomes. Moreover, it is crucial that there is a universal screening for mothers' mental state and psychological distress to manage any potential post-traumatic stress disorder and feelings of shock, depression, or anxiety that may be occurring [39]. Additionally, although we see a lot of evidence on the effects of a diagnosis on the mother, there is still research that needs to be done on the effects on fathers and siblings of the diagnosed child. Overall, the father and siblings also have a large impact on the child’s life and long-term outcomes, so it is important to explore this connection as well.

## Conclusions

Fetal echocardiography plays a critical role in early detection and management of congenital heart disease, with increasing evidence supporting its use even in the late first trimester. While early imaging enables identification of major structural abnormalities and improves perinatal planning, its limitations should also be considered. This includes reduced sensitivity for mild CHDs and operator dependence. Therefore, continued follow-up and routine screening are necessary in the second trimester. Advances in artificial intelligence and imaging technologies offer a promising future to enhance detection accuracy, standardize protocols, and reduce variability. Prenatal diagnosis not only influences postnatal outcomes through increased preparedness and targeted interventions but also impacts family decision making and the mother’s physiological well-being. Moving forward, refining early screening techniques, expanding access, and maintaining safety are important in improving clinical outcomes in fetal cardiology.

## Key Points

- Congenital Heart Disease (CHD) is the most common birth defect (8-9.4 per 1,000 births) and is a major contributor to neonatal morbidity and mortality.
- Early fetal echocardiography (11-15 weeks) allows earlier detection of cardiac abnormalities compared to the traditional 18–22-week scan, enabling prenatal planning and intervention.
- Early diagnosis of CHD improves outcomes by enabling delivery at specialized centers, immediate neonatal care, and informed clinical decision making.
- Early diagnosis most strongly detects major CHDs such as hypoplastic left heart syndrome and tetralogy of Fallot, while mild defects are often missed and require second trimester follow up.
- Limitations of early fetal echocardiography include small fetal heart size, rapid heart rate, incomplete structural maturation, and reduced visualization of fetal anatomy.
- Maternal (BMI, fibroids, scars) and fetal factors (position, gestational age) impact image quality and diagnostic accuracy.
- Artificial Intelligence (AI) enhances fetal echocardiography by improving image quality, automating cardiac views, reducing operator dependence, and achieving high diagnostic sensitivity.
- Limitations of AI include reliance on high quality data, integration challenges, ethical considerations, and preventing full clinical replacement of physicians.
- Prenatal CHD diagnosis has psychological impact on families, including increased anxiety and stress, highlighting the need for counseling and support systems.
- Future advancements should focus on combining AI tools with standardized early fetal echocardiography to improve consistency and expand access to early CHD detection.
